# Prevalence and Correlates of Food Insecurity among Palestinian Refugees in Lebanon: Data from a Household Survey

**DOI:** 10.1371/journal.pone.0130724

**Published:** 2015-06-22

**Authors:** Hala Ghattas, AnnieBelle J. Sassine, Karin Seyfert, Mark Nord, Nadine R. Sahyoun

**Affiliations:** 1 Center for Research on Population and Health, American University of Beirut, Beirut, Lebanon; 2 Department of Economics, School of Oriental and African Studies, London, United Kingdom; 3 Economic Research Service, USDA, Washington, DC, United States of America; 4 Department of Nutrition and Food Science, University of Maryland, College Park, MD, United States of America; Wageningen University, NETHERLANDS

## Abstract

Lebanon hosts the highest per capita refugee concentration worldwide. The Palestinian presence in Lebanon dates from 1948 and they remain a marginalized population. No information on their food security status has been reported previously. A survey of a representative sample of Palestinian refugee households in Lebanon (n = 2501) was conducted using a stratified two stage cluster sampling approach. We measured food insecurity using a modified USDA household food security module, locally validated. We collected data on household demographic, socioeconomic, health, housing, coping strategies and household intake of food groups and analysed these by food security status. About 41% (CI: 39-43) of households reported being food insecure and 20% (CI: 18-22) severely food insecure. Poor households were more likely to be severely food insecure (OR 1.41 (1.06-1.86)) while higher education of the head of household was significantly associated with protection against severe food insecurity (OR 0.66 (0.52-0.84)). Additionally, higher food expenditure and possession of food-related assets were significantly associated with food security (OR 0.93 (0.89-0.97) and OR 0.74 (0.59-0.92), respectively). After adjusting for confounders, households where at least one member suffered from an acute illness remained significantly more likely to be severely food insecure (OR 1.31(1.02-1.66)), as were households whose proxy respondent reported poor mental health (OR 2.64 (2.07-3.38)) and poor self-reported health (OR 1.62 (1.22-2.13). Severely food insecure households were more likely to eat cheaper foods when compared to non-severely food insecure households (p<0.001) and were more likely to rely on gifts (p<0.001) or welfare (p<0.001). They were also more likely to have exhausted all coping strategies, indicating significantly more frequently that they could not do anything (p = 0.0102). Food insecurity is a significant problem among Palestinian refugees in Lebanon and is likely to be exacerbated at this time when the Syrian crisis amplifies the problem.

## Introduction

The Palestinian presence in Lebanon dates from 1948 and is one of protracted (long term) refugee status rather than refugees fleeing from recent conflict. In Lebanon, there were 436,154 Palestinian refugees registered in 2010 with the United Nations distributed over 12 camps and segregated gatherings, which are communities outside official camps [1].

Despite their longstanding presence in Lebanon, Palestinian refugees remain a marginalized population excluded from key aspects of social, political and economic life in the country[2]. These refugees require special work permits that are very hard to obtain and are restricted to certain types of non-professional employments. Refugees are prohibited from running a business, owning a property and have limited civil rights and representation. They depend mostly on humanitarian assistance from the United Nations Relief and Works Agency for Palestine Refugees in the Near East (UNRWA) which provides welfare, education and health services [3]. The absence of rights and social isolation of Palestinian refugees makes them vulnerable to inadequate access to health care, education, food and they may consequently experience food insecurity.

Food security is defined by the World Food Summit 1996 as “when all people at all times have sustainable physical and economic access to sufficient, safe and nutritious food, to meet their dietary needs and food preferences, for a healthy and productive life”[4]. Food insecurity which is the absence of one or more of these conditions is an underlying cause of malnutrition which may have an impact on health status and impair mental and physical development.

The status of food security among Palestinian refugees in Lebanon has not been reported previously. Yet, an assessment of the food security of this vulnerable population is essential because of the potential consequences on its public health status and the implication on policy and food aid decisions. In 2010, commissioned by UNRWA, we undertook a study to describe the living conditions and health and food security status of Palestinian refugees. This paper presents the prevalence of food security in a representative sample of Palestinian refugees residing in camps and in gatherings in Lebanon, and identifies the socio-economic and dietary correlates of food insecurity in this population.

## Materials and Methods

### Study Design and Sampling

#### Sampling approach

Data collection took place in July-August 2010. A stratified multi-stage cluster sampling approach was used. The survey was carried out in all 12 Palestinian camps and in 20 gatherings in five administrative areas of the country. Clusters were defined as distinct Palestinian refugee neighbourhoods; either camps or unofficial gatherings. All camps were sampled, and 20 of the 187 gatherings in the country were randomly selected. Each gathering cluster was sampled proportionate to estimated population size. Within camps and gatherings, households were selected randomly according to a specified algorithm that data collectors had been trained in.

About 2,575 households were eligible and 2,501 consented to be interviewed (97.1% response rate). On identifying a household, data collectors assessed eligibility and sought consent. Face-to-face interview with a proxy adult respondent from the household, preferably a senior female member responsible for food preparation, were carried out. Indeed, 82% of proxy respondents were women.

The study design was approved by the American University of Beirut Institutional Review Board (IRB). Consent forms and questionnaires were rendered in Levantine Arabic dialect. A waiver of written consent was approved by the IRB and oral consent was sought, as data were collected on illegal work activities therefore placing individuals at risk of prosecution. Respondents were given a copy of the consent form for their own reference, and were read the information and given the opportunity to ask questions. Data collectors signed a statement indicating that the respondent voluntarily agreed to participate in the study and that she/he is aware that she/he may discontinue participation at any time. In case the participant was illiterate, a witness signature was required.

#### Data collection questionnaire

The questionnaire included a household roster with questions on individuals’ age, marital status, education, employment, and illness. All physical health characteristics were proxy respondent reported conditions. For acute illnesses, a recall period of six months prior to the interview was used. Data on household characteristics such as household income, expenditure, assets, and welfare assistance were also collected. A food-related asset scale was derived based on the ownership of refrigerator, freezer, oven, and microwave, each contributing one point to the scale.

Poverty was based on household consumption expenditure equivalent to minimal food and non-food livelihood requirements per adult equivalent and was evaluated against the inflation adjusted poverty line to infer prevalence of poverty among refugees [5]. The poverty line used was $6 per day.

Mental health of proxy respondents was assessed using the five-item Mental Health Inventory (MHI-5) [6] which is widely used in surveys of general health and a good predictor of depression. High scores indicate good mental health. We used the more conservative MHI-5 cut-off point of 52, since it yields lower prevalence of poor mental health compared to higher cut-offs [7,8].

Questions on coping mechanisms adapted from the Coping Strategies Index were also administered [9]. These questions inquired about whether the respondents or other members of the household did one or more of the following actions: worked more to obtain food, borrowed money, borrowed food, ate cheaper food, accepted gift/donation or could do nothing about it. The answers were coded as yes or no.

Food insecurity in this study was measured as household experience of food insecurity, which includes components of worry about running out of food, reduced food quality and reduced quantity. Household food security was assessed using a 6 question food security scale. The questions were derived from the USDA food security survey module [10,11] and the Yemeni Food Security Questionnaire [12]. The module was internally validated using statistical methods based on the Rasch measurement model. This model is used to assess the psychometric characteristics of the questionnaire items and the extent to which they measure the same underlying latent trait, in this case, the severity of food insecurity. A detailed explanation of the development of the module, as well as the validation process has been published elsewhere [13]. Positive responses were counted as one point and households were classified according to the total score: Food secure (0–1), moderately food insecure (2–4), and severely food insecure (5–6).

The food section also included a household food frequency module which measured frequency per day, week or month of household consumption of the food groups: meat, dairy, fruits, vegetables, pulses, soda and sweets. Responses were converted to weekly consumption.

### Statistical Analysis

Both, univariate and multivariate analyses (of the survey data were performed using Stata version 12 (StataCorp) and the STATA svyset command to adjust for sampling design and selection probabilities in the analysis. Entire cases with items missing were deleted and considered non-response.

Proportions and percentages displayed are weighted estimates. Adjusted Wald and *F* tests were used to test for significant differences between groups. Associations between socio-economic and health characteristics and severe food insecurity were assessed using logistic regression. Stepwise logistic regression analysis was used in the multivariate model to select predictors of food insecurity. Variables were excluded from the model if they were collinear with other variables or if they did not improve the fit of the model. Food consumption data were normalised using log-transformation in the form of ln(x+1) to account for null values, and back-transformed into the original scale using exponential transformation for the adjusted mean consumption of food groups.

## Results

Though 2501 respondents consented to be interviewed and at least partially completed the questionnaire, the complete food security module was available from 2493 households. In total, 38% (CI: 36–41) of all households reported being food secure, 41% (CI: 39–43) moderately food insecure and 20% (CI: 18–22) severely food insecure.

Overall, 64% of Palestinian refugee households lived in camps versus gatherings and a larger percentage of the households at risk of food insecurity lived in camps. Additionally, 46% of head of households were illiterate or did not complete primary education and 41% were unemployed. These demographics also showed that food insecurity was more prevalent among households whose head had lower education or was employed in a low skilled job (Table 1).

**Table 1:** Characteristics of Palestinian refugees by levels of household (HH) food security.

	n	Food secure (scores 0–1)	Moderately food insecure (scores 2–4)	Severely food insecure (scores 5–6)	Total
**HH Demographics**					
HH size, mean (95% CI)	2493	4.2 (4.0–4.36)	4.6 (4.45–4.76)	4.7 (4.40–5.0)	4.45 (4.3–4.6)
Number of children under 15 years of age, mean (95% CI)	2493	1.05 (0.90–1.20)	1.29(1.18–1.39)	1.24(1.09–1.39)	1.18(1.09–1.28)
Living in camps versus gatherings, %	2493	56.3	67.4	70.5	63.7
**Head of HH Demographics**					
Female head of HH, %	2462	20.9	23.6	25.6	23.0
Head of HH education attainment, %	2493				
Illiterate/incomplete primary		38.1	47.1	58.2	45.9
Completed primary level		30.4	26.3	21.4	26.9
Above primary level (including middle-, high- school and college)		31.5	26.6	20.4	27.3
Unemployed head of HH versus employed, %	2466	37.8	41.9	47.2	41.4
Head of HH occupation, %	1330				
Elementary		15.6	27.5	36.6	24.2
Crafts		48.8	48.5	50.6	49.0
Service		18.6	15.8	10.7	16.0
Associate professional		3.3	1.6	0.8	2.1
Professional		13.7	6.6	1.4	8.6
**HH Socio-economic factors**					
Poor, %	2493	45.2	64.8	72.2	58.7
Average monthly food expenditure per capita (U.S. dollars), mean (95% CI)	2402	81.0 (75.2–86.8)	63.0 (60.4–65.6)	54.8 (49.6–60.1)	68.4 (65.2–71.6)
Number of food-related assets ^a^, mean (95% CI)	2477	2.3 (2.24–2.33)	2.1 (2.08–2.18)	2.0 (1.93–2.09)	2.17 (2.12–2.21)
Receiving welfare, %	2493	31.9	50.6	57.0	44.6
**HH Health** ^**b**^					
At least one member reports acute illness in HH, %	2469	48.8	62.5	67.1	58.1
At least one member reports chronic illness in HH, %	2469	66.7	80.4	83.8	75.7
At least one member reports disability in HH, %	2469	12.1	17.6	24.4	16.9
Mental health inventory (MHI-5) score of respondent <52/100, %	2493	21	35.7	56.7	34.2
Respondent’s self-rated health, %	2482				
Very good/good		41.6	21.3	17	28.3
Fair		38.1	43.6	39.3	40.6
Not good/very bad		20.4	35.1	43.7	31.3

Estimates are weighted percentages or mean values and 95% confidence intervals.

^a^ Food-related assets represent the sum of fridge, freezer, oven, and microwave.

^b^ All health characteristics are self-reported physician diagnosed illnesses within the last six months.

More than half of the population were considered poor (58.7%) and 72% of the households experiencing severe food insecurity were poor and tended to have lower food expenditure than food secure households. Total monthly household expenditure was significantly lower in severely food insecure households as compared to food secure households, indicating that availability of money is an issue. However, money allocation to food was also slightly but significantly reduced in the severely food insecure, with 36.6% of total expenditure spent on food as compared to 40.2% in the food secure (p = 0.02). Also, food secure households had more food related assets (refrigerator, freezer, oven, and microwave). The decrease in the number of food related assets and food expenditure was monotonic across the three categories of food insecurity. Half or more of the food insecure households were receiving welfare but yet this did not appear sufficient to allay the response to food insecurity.

Indicators of physical and mental health were significantly associated with food security. Severely food insecure households were more likely to self-report not good/very bad health and to have at least one member suffering from a chronic illness (75.7%) or a disability (16.9%). They were also more likely to have a member who suffered from an acute illness during the six months preceding the survey (58.1%). Proxy respondents from severely food insecure households scored lowest in the mental health module, indicating worse mental health (Table 1).

Results of the univariate logistics regression analysis in Table 2 show that household size was significantly associated with severe food insecurity and so was living in camps versus gatherings. However, these variables were no longer significant in the multivariate logistic regression after controlling for confounders. Although a higher proportion of food insecure and severely food insecure households were headed by women than food secure households, the association between food insecurity and gender of head of household was not statistically significant.

**Table 2:** Logistic regression model for predictors of severe food insecurity among Palestinian refugee households in Lebanon.

	Severely food insecure			
Predictors	Crude OR ^a^ (95% CI)	P-value	Adjusted OR ^b^ (95% CI)	P-value
**HH Demographics**				
Household size	1.06 (1.01–1.12)	0.018		
Number of children under 15 years of age	1.04 (0.96–1.12)	0.38		
Living in camps (referent: living in gatherings)	1.46 (1.11–1.92)	0.006		
**Head of HH Demographics**				
Female head of HH (referent: male)	1.20 (0.95–1.51)	0.119		
Head of HH educational attainment (referent: Did not complete primary level)				
Completed primary level	0.55 (0.40–0.78)	0.001	0.63 (0.44–0.90)	0.012
Completed any level above primary	0.52 (0.39–0.69)	0.000	0.66 (0.49–0.88)	0.005
Head of HH employed (referent: not employed)	0.74 (0.60–0.91)	0.005		
**HH Socio-economic Factors**				
Poor (referent: not poor)	2.10 (1.63–2.69)	0.000	1.41 (1.06–1.86)	0.017
Food expenditure/capita/HH (per $10)	0.91 (0.87–0.95)	0.000	0.93 (0.89–0.97)	0.000
Food-related assets	0.61 (0.48–0.77)	0.000	0.74 (0.59–0.92)	0.008
Receiving welfare	1.87 (1.42–2.46)	0.000		
**HH Health**				
At least one member reports chronic disease	1.85 (1.38–0.48)	0.000		
At least one member reports disability	1.84 (1.30–2.60)	0.001		
At least one member reports acute illness	1.61 (1.27–2.05)	0.000	1.31 (1.02–1.66)	0.031
Mental health inventory (MHI-5) score of respondent <52/100 (referent: ≥52/100)	3.28 (2.61–4.13)	0.000	2.64 (2.07–3.38)	0.000
Respondent’s self-rated health (referent: very good/good)				
Fair	1.76 (1.30–2.38)	0.000	1.28 (0.96–1.71)	0.088
Not good/ Very bad	2.86 (2.2–3.71)	0.000	1.62 (1.22–2.13)	0.001

^a^ Estimates are weighted odds ratios generated from univariate logistic regression in relation to severe HH food insecurity.

^b^ Estimates from multivariate logistic regression model using stepwise selection (n = 2357) are weighted OR in relation to severe HH food insecurity. Model statistics include goodness-of-fit test: F (9, 1389) = 0.38, P = 0.9429.

Poor households were twice as likely to be severely food insecure and this variable remained significant though attenuated in the larger model (OR 1.41 (1.06–1.86)) while higher education of the head of household was significantly associated with protection against severe food insecurity (OR 0.66 (0.52–0.84)). Additionally, higher food expenditure and possession of food-related assets were significantly associated with food security and remained significant in the larger model (OR 0.93 (0.89–0.97) and OR 0.74 (0.59–0.92), respectively.

The presence of a household member with a health condition such as an episode of acute illness within six months of the interview, a chronic condition and/or a disability was associated with a higher risk of severe food insecurity, however, only households where at least one member suffered from an acute illness remained significantly more likely to be severely food insecure (OR 1.31(1.02–1.66)), as were household whose proxy respondent reported poor mental health (OR 2.64 (2.07–3.38)) and poor self-reported health (OR 1.62 (1.22–2.13)).

There was a significant decline in mean intake of meat, dairy, fruits and vegetables from food secure to moderately and severely food insecure households (Table 3). This was also true for soda and sweets intake. Pulses are the only food group that was consumed in significantly higher amounts with an increase in food insecurity status.

**Table 3:** Mean dietary intake of food categories by Palestinian refugee households and by levels of food security.

Food Groups	n	Food secure (scores 0–1)	Food insecure (scores 2–4)	Severely Food insecure (scores 5–6)
Meat	2488	2.6^a^ (2.5–2.7)	1.5^b^ (1.4–1.55)	0.9^c^ (0.8–0.97)
Dairy	2489	6.4^a^ (6.0–6.8)	5.0^b^ (4.8–5.2)	3.5^c^ (3.2–3.9)
Fruits	2489	5.4^a^ (5.1–5.7)	2.7^b^ (2.5–2.8)	1.6^c^ (1.4–1.7)
Vegetables	2488	6.5^a^ (6.3–6.6)	5.0^b^ (4.8–5.3)	4.2^c^ (3.8–4.6)
Pulses	2488	2.1^a^ (1.95–2.2)	2.3^b^ (2.1–2.4)	2.6^c^ (2.4–2.8)
Soda	2484	3.5^a^ (3.2–3.9)	2.2^b^ (2.1–2.4)	1.9^c^ (1.7–2.1)
Sweets	2484	2.6^a^ (2.4–2.9)	2.1^b^ (1.9–2.3)	1.6^c^ (1.3–1.8)

Estimates are weighted means and 95% confidence intervals. P-values are obtained using one-way analysis. The data on food category consumption was log transformed using the equation ln(x+1) and back transformed to achieve a normal distribution.

^a^ Significantly different from moderately and severely food insecure households (p<0.05).

^b^ Significantly different from food secure and severely food insecure households (p<0.05).

^c^ Significantly different from food secure and moderately food insecure households (p<0.05).

Coping mechanisms of severely food insecure households were assessed (Table 4). Severely food insecure households were less likely to eat the same quantities of cheaper foods when compared to non-severely food insecure households (p<0.001) and were more likely to rely on gifts or welfare (p<0.001). They were also more likely to have exhausted all coping strategies, indicating significantly more frequently than non-severely food insecure households that they could not do anything (p = 0.0102).

**Table 4:** Coping mechanisms employed by Palestinian refugee households who reported that the food that they had did not last, and they did not have money to buy more.

Coping mechanism	Total	Non-severely food insecure (scores 0–4)	Severely food insecure (scores 5–6)	P-value
Ate less quantity of normally eaten food, %	21.6	20.5	23.8	0.23
Ate same quantity of cheaper food, %	39.5	47.4	25.1	0.000
Reduced price and quantity, %	19.6	19.7	19.3	0.86
Received gift/help, %	8.2	5.2	13.7	0.000
Borrowed food, %	5.2	4.3	6.9	0.07
Could not do anything, %	2.8	1.7	4.9	0.01

Values are percentages; categorical data were compared using Pearson’s chi-squared. Questions on coping mechanisms were asked for respondents who reported that the food they had did not last, and they did not have enough money to buy more food. Sample size is therefore 1371 for all coping mechanisms.

## Discussion

This study finds that nearly two thirds of Palestinian refugee households living in Lebanon were food insecure (63%) in 2010. Twenty percent of these household were severely food insecure, and in 15% of households, at least one member of the household had spent a whole day without food or gone to sleep hungry due to lack of food or insufficient money to buy food, in the six months preceding the interview. These results are supported by lower intake of all food groups, except for pulses. This is a population at particular risk of experiencing repeated bouts of scarcity that could lead to chronic malnutrition.

In this study, food insecure families were poorer and headed by individuals who mostly had low educational attainment, were underemployed or unemployed and reported worse household health conditions. Although households living in camps were more likely to be food insecure, this association was no longer significant when adjusted for other socio-demographic variables. This is likely due to the confounding effect of poverty and living conditions; in fact, 66% of households living in camps were classified as poor as compared to 45% of those living outside of camps, and Palestinian camps in Southern Lebanon had particularly poor housing conditions, and overcrowding.

In Lebanon, restrictive policies on employment and education are added stressors contributing to poverty and poor living conditions [14]. In fact, Palestinians have very poor housing conditions [15], with a mean crowding level of 1.76 persons per room, and high levels of multi-morbidity [16].

There was a high prevalence of poor health conditions among the refugee population in our study, and more particularly among the food insecure households. Food insecurity is characterized by reduction in food expenditure, reduced food intake, and changes in quality of food consumed, whereby diet diversity can be decreased and consumption of energy-dense foods increased[17]. These energy-dense foods, including refined grains, added sugars, and added saturated fats tend to be of poor nutritional quality and are less expensive than nutrient-dense foods[18]. Our results show that food insecure households have compromised diet quality, and consume less weekly servings of fruits, vegetables, dairy, and meat thus lower levels of micronutrients, including the B complex vitamins, magnesium, iron, zinc, and calcium.

These dietary patterns are linked to the development of chronic disease, including hypertension, hyperlipidemia, and diabetes, and these micronutrient deficiencies can be associated with higher susceptibility to acute infections, especially among children. In fact, more than half of the households in this study had at least one member with an acute illness over the six months preceding the interview, and acute illness remained significantly associated with severe food insecurity after adjusting for other socio-demographic correlates.

Certainly poor living conditions and crowding have an exacerbating effect on the health status of the residents. This may lead to time lost from work and reduced income. Chronic health conditions were also quite prevalent but this may be due to extended families with older adults living together in the same household. Nevertheless, household members who are unhealthy may be an additional burden on families that have to spend money on medications at the expense of other necessities including food. Additionally, a third of the survey respondents reported depressive symptoms and this was significantly associated with food insecurity. Depression and poor health status may result in lower employment and income, therefore contributing to poverty and food insecurity but may also be an outcome of food insecurity, poor living conditions and insufficient resources. Due to the cross-sectional nature of this study, we were unable to establish the direction of this association between illness and food insecurity.

The households in our study resorted to several coping strategies such as eating less or eating cheaper food, borrowing or receiving money or food, however, about 5% of the severely food insecure respondents report that they could not do anything about their food situation.

Lebanon hosts the highest per capita refugee concentration worldwide [19]. Palestinian refugees are the oldest, followed by Iraqi and then Syrian. The war in Iraq has led to waves of refugees in Lebanon. Funded by the United Nations High Commissioner for Refugees (UNHCR), a study was conducted in Lebanon which showed that only 20% of the Iraqi refugees were food secure [20]. More recently, the Syrian civil war has resulted in more than one million Syrians being displaced to Lebanon[21]. As of April 2014 over 53,000 Palestinian refugees from Syria migrated to Lebanon in search of safety [3] and approximately 51% of them are now hosted in Palestinian refugees in the camps in Lebanon[22], posing additional burden on camp households which were already more likely to be poor, and food insecure, and on the UNRWA infrastructure such as the welfare program, schools and health care system, potentially extending resources even further from the original camp residents.

This paper highlights that the correlates of food insecurity in this population are poverty, illness and low educational attainment. Although UNRWA provides education and food, these are not sufficiently alleviating poverty and hence food insecurity, most probably due to strict government policies that restrict the right of Palestinians to generate income, and serve as a disincentive for continuing education. Although this study shows that food insecure households are more likely to receive food assistance and welfare, this appears to be insufficient to lift the whole population out of food insecurity. Crowding and poor living conditions also possibly offset the positive effects of UNRWA’s health services, and have been associated with multi-morbidity.[16]

This study presents data pre-Syria conflict, and highlights the precarious condition of Palestinian refugees living in Lebanon, who suffer from substantial rates of food insecurity. The influx of Palestinian refugees from Syria is likely to further exacerbate the living conditions and food insecurity of Palestinian refugees previously in Lebanon. Food insecurity can result in chronic malnutrition and poor health outcomes which have long-term consequences. This implies that targeting food aid to the most food insecure will continue to be an essential part of UNRWA’s programs. Our results also emphasize the need for continued funding support to UNRWA’s services to provide assistance to this vulnerable population. The root causes of poor health and food insecurity need to be addressed by increasing funding towards housing and infrastructure, reforming policies to increase the rights of this vulnerable population, and finding a permanent solution to the plight of the Palestinian refugee population.
